# The “Unlinkables”: A Case Series of Overcoming Social Determinants of Health for Successful Linkage to Care for HIV from the ED

**DOI:** 10.5811/cpcem.39649

**Published:** 2025-07-29

**Authors:** Phillip Moschella, Mirinda Ann Gormley, Kiran Faryar

**Affiliations:** * Prisma Health, University of South Carolina, School of Medicine Greenville, Department of Emergency Medicine, Greenville, South Carolina; † Clemson University School of Health Research, Clemson, South Carolina; ‡ University Hospitals Cleveland Medical Center, Case Western Reserve University School of Medicine, Department of Emergency Medicine, Cleveland, Ohio

**Keywords:** HIV, linkage to care, emergency department, social determinants of health

## Abstract

**Introduction:**

Despite the success of emergency department (ED)-based universal HIV screening programs in select cities, widespread integration of similar programs across the United States has not followed. Within the US Centers for Disease Control and Prevention (CDC)-designated “Ending the HIV Epidemic (EHE)” areas, ED-based HIV screening is low. This case series highlights successful strategies for notification and linkage to care of patients with various challenging social determinates of health (SDoH). The goal is to inspire more EDs to offer universal HIV screening by providing insight into these challenging SDoH and successful strategies to overcome them.

**Case Series:**

We describe four cases, two from a site in upstate South Carolina and two from Cuyahoga County in Ohio, that highlight successful notification and linkage to care of these perceived “worst-case” scenarios. Both ED-based programs are located in CDC-designated EHE areas. We discuss ED screening opportunities and successful linkage for these minority patients (21–36 years of age), and highlight the concomitant and challenging mental health and substance use disorders, and SDoH that were overcome. All four of these patients are currently receiving treatment for HIV and 3 of the 4 have reached viral suppression.

**Conclusion:**

Despite challenging SDoH including unstable housing and lack of transportation, phone, and even legal identification documentation, these ED-identified patients with HIV were successfully notified of their disease status and linked to care. The patient navigators used perseverance, connections to local community resources, and leveraged family support to achieve linkage success. The cases serve as both a roadmap and source of inspiration to other EDs in priority EHE areas to begin ED HIV screening programs.

## INTRODUCTION

Over a decade of literature has demonstrated that emergency departments (ED) can systematically identify and successfully link patients with HIV to long-term care, yet few articles have described the coexisting mental health and substance use disorders, and social determinants of health (SDoH) barriers that make successful linkage to care difficult for patients with either newly or previously diagnosed HIV who present to an ED.^1^,^2^ This case series demonstrates how these coexisting disorders and SDoH posed challenges to linkage to HIV treatment for four patients from two different healthcare systems. Patients were identified as having experienced the most barriers to linkage to care by research coordinators within each program. These programs were located within priority US Centers for Disease Control and Prevention (CDC)-designated “Ending the HIV Epidemic” (EHE) jurisdictions (Cuyahoga County, Ohio and Greenville County, South Carolina).

The EHE initiative is a nationwide program coordinated by the US Department of Health and Human Services working with the CDC on community-specific programs and collaborations to reduce new HIV infections and support treatment and prevention. The EHE initiative has identified 50 local areas and seven entire states as priority jurisdictions where more than half of new HIV diagnoses occur.^3^ We highlight the following complicating medical comorbidities:

1. mental health disorders such as depression, anxiety, bipolar disorder, schizophrenia, or antisocial personality disorder; and
2. substance use disorder including use of opioids, stimulants, alcohol, hallucinogens, or any other abused prescriptions or illicit substances. We also highlight various SDoH that complicate linkage including transportation issues, lack of reliable contact information, current or prior incarceration, and housing insecurity.

This case series highlights the challenges surrounding several medical comorbidities and the SDoH that complicated or delayed successful linkage to care across two separate healthcare systems, each located in an EHE priority jurisdiction, to emphasize the unique and shared challenges faced across the US (Table 1). These ED patients represent some of the most challenging and medically and socially complex patients with HIV to provide insight and inspiration for other EDs that successful linkage can be obtained.

## SETTINGS AND PROGRAMS

Site 1 is in an urban, academic Level I trauma center in Greenville County, South Carolina. Since August 2019, Site 1 has implemented a universal, ED-based opt-out HIV-screening program with linkage to care for eligible adults (≥18 years of age). Adults are eligible for screening if they have no recorded history of HIV infection and have not been screened within the prior 12 months. The program uses a serum HIV 1/2 antigen and antibody (Ag/Ab) immunoassay with HIV 1/2 Ab differentiation and RNA quantitation as confirmatory tests for HIV diagnosis. People with HIV not in care may be identified through this program. These individuals may have a prior positive HIV Ag/Ab screen and confirmatory test or HIV RNA viral load and no documented antiretroviral medication listed in our electronic health record (EHR) and are not currently in care. All those testing positive for HIV through this program are contacted and confirmed in treatment by full-time patient navigators.

Those newly diagnosed or patients already diagnosed with HIV but not in care are linked to an infectious disease clinic within the hospital system or one of two non-profit clinics, which partner with the program. Between January 1, 2023 December 31, 2024, this program performed a total of 50,438 HIV tests (2023: 20,866 tests; 2024: 29,572 tests), approximately 2,100 per month, with a positivity rate of 0.33%. Most of those who screened positive for HIV were non-Hispanic Black (54.3%) and male (79.8%) with an average age of 43 years. Of the 164 individuals who tested positive, 54 (32%) were confirmed to already be in care. Of the remaining 110 HIV-positive individuals, 95 (86.4%) were linked to treatment, 3 (2.7%) were deceased at time of follow-up, and only 12 (10.9%) are currently lost to follow-up. Of the 95 individuals who were linked to treatment, 71 (74.7%) were newly diagnosed and 24 (24.3%) were previously diagnosed but not in care.

*CPC-EM Capsule*

What do we already know about this clinical entity?

*The HIV epidemic continues across the US. The ED represents a unique and critical venue for HIV screening and linkage to care*.

What makes this presentation of disease reportable?

*Many EDs providing HIV screening may not be aware of barriers posed by social determinants of health (SDo,) which prevent patients with HIV from accessing treatment*.

What is the major learning point?

*Although ED patients may face SDoH barriers to accessing HIV treatment, overcoming barriers is possible using ED adjunct staff and a sustained linkage-to-care approach*.

How might this improve emergency medicine practice?

*Emergency clinicians and staff can assist in identifying and overcoming patient SDoH barriers to successfully link patients to HIV treatment*.

Site 2 is in an urban, academic Level I trauma center in Cuyahoga County, Ohio. Since August 2022, Site 2 has implemented an opt-out universal HIV screening program using clinician-, patient navigator-, and EHR-initiated approaches. The primary ED at Site 2 uses a serum HIV 1/2 Ag/Ab immunoassay with HIV 1/2 Ab differentiation confirmatory test for HIV diagnosis. Patients with HIV who are not in care are identified by a prior positive HIV Ag/Ab screen and confirmatory test or HIV RNA viral load and no documented antiretroviral therapy listed in the EHR or by self-report. Site 2 has an associated infectious disease clinic specifically for patients with HIV; those newly diagnosed with HIV as well as those with HIV not in care are referred to the clinic via warm hand-off by two full-time patient navigators. Of 1,960 HIV tests performed between 2022–2024, 64 (3.3%) were positive. Of those who screened positive for HIV, most were non-Hispanic Black 52 (81.3%) and male 38 (59.4%). Of the 64 individuals who tested positive, 17 were identified as newly diagnosed active infections, and 12 (70.6%) of those newly diagnosed were linked to treatment.

## CASE SERIES

### Case 1

A 36-year-old non-Hispanic Black male presented to the ED at Site 1 10 times between January 2022–April 2024 for various chief complaints. He screened positive for HIV in January 2022 and was ultimately linked to HIV care in May 2023. As a known individual with HIV out of care who frequented this ED, across his various ED visits, repeat HIV screening was not obtained. The time from the first ED patient engagement with our patient navigator (September 2022) to a successful follow-up appointment with initiation of antiretroviral therapy was 235 days. The patient had multiple medical comorbidities that complicated his successful linkage including schizophrenia, and alcohol and cocaine use disorders. These comorbidities contributed to various complicating SDoH including unstable housing, multiple episodes of incarceration in 2018 and 2024, and his lack of transportation and phone.

The navigators specifically highlighted lack of transportation, chronic uncontrolled pain due to medication noncompliance, and “couch surfing” between family members as the main barriers to linkage. The navigators used various strategies including repeated re-engagement during subsequent ED visits, appointment reminder calls 1–2 days before his scheduled appointments, and finally arranging unfunded transportation using his family and social network. He is currently in care but has not yet reached viral suppression.

### Case 2

A 21-year-old non-Hispanic Black male presented to the ED at Site 1 in August 2023 with concerns for a sexually transmitted infection (STI). HIV testing was included in his STI workup. His HIV screening was positive, but confirmation testing was not completed secondary to a lack of blood sample. Patient navigators successfully contacted the patient, and he returned to the ED for a second visit where his confirmation testing and HIV post-test counseling were completed. The time from ED diagnosis and engagement to successful outpatient linkage and initiation of antiretroviral therapy was 20 days. He had multiple comorbidities including depression, anxiety, and post-traumatic stress disorder secondary to past physical abuse from various family members and domestic partners. His SDoH barriers included unstable housing, food insecurity, and lack of transportation. His linkage was complicated most by his housing insecurity, specifically needing to leave his current partner’s house due to domestic violence. Throughout 2023 and early 2024, navigators in both the ED and infectious disease clinic remained in close contact with the patient and connected him to community resources including housing and electricity support to facilitate his safe transition to living independently. Despite his challenges with his living situation, he has been compliant with treatment and his HIV viral load was undetectable as of January 2024.

### Case 3

A 35-year-old non-Hispanic Black male presented to and subsequently eloped from the ED at Site 2 in early September 2023. Blood work was obtained prior to elopement that showed both lymphopenia and thrombocytopenia. The patient was unable to be contacted to discuss results, but he returned to the Site 2 ED later that month and was diagnosed with methamphetamine-induced psychosis and held for psychiatric evaluation. During this visit, based on his previous lab work and as part of the medical evaluation for new-onset psychosis, he tested positive for HIV. The patient was transported to a psychiatric facility with post-test counseling completed prior to his transfer. Subsequent HIV ribonucleic acid (RNA) confirmatory testing was delayed secondary to multiple factors but was obtained in November 2023 and he was successfully linked to care in December 2023. The time from ED diagnosis and engagement to successful outpatient linkage and initiation of antiretroviral therapy was 77 days.

The patient had multiple comorbidities including schizophrenia, methamphetamine-induced psychosis, and methamphetamine use disorder. He had multiple admissions to various substance use disorder- and psychiatric treatment facilities across the state that ultimately delayed his linkage to HIV care. The patient navigators noted several key barriers surrounding SDoH including unemployment, lack of health insurance, and transient housing. Despite these barriers, he had strong family connections and requested that family be informed of his diagnosis to help with linkage to care. The navigators coordinated follow-up appointments and transportation through his family members, whom he lived with intermittently. After successful linkage to HIV care, the patient is now living with one family member who helps with transportation. He has been compliant with treatment and reached viral suppression in March 2023.

### Case 4

A 33-year-old non-Hispanic Black male presented to the ED at Site 2 in September 2023 with a chief complaint of “anxiety.” As part of our ED-based HIV screening program, he was screened and found positive for HIV with reflex confirmation testing. The patient eloped from the ED before post-test counseling could be completed. He was unable to be contacted upon discharge but returned to the same ED in October 2023. At that visit, both his post-test counseling and linkage to care were completed, and he was seen the same day at a clinic across the street from the ED. His time from HIV diagnosis to initiation of treatment was 20 days.

The patient has multiple comorbidities including attention-deficit/hyperactivity disorder, anxiety, bipolar 1 disorder, depression, and schizophrenia. He had been incarcerated several times and had multiple SDoH that complicated his linkage to care to outpatient office visits. Specifically, the patient navigators noted his lack of housing compounded by a lack of a legal identification documentation, which prevented him access to local shelters. The patient did begin living in a tent behind a fast-food restaurant and could be contacted there intermittently using a Wi-Fi messaging service on a shared phone and through intermittent direct contact over a meal at the restaurant. He has been compliant with treatment and reached viral suppression in February 2024.

## DISCUSSION

This case series is the first to discuss the pathway to successful linkage to care for four patients with HIV from the ED with various complicating mental health or substance use disorder comorbidities and SDoH. The intersection of these complicating factors often dissuades clinicians from offering HIV screening. We highlight the journey within priority jurisdictions of the EHE campaign representing areas of the country that are driving the current HIV epidemic. These selected patients all had significant medical comorbidities or SDoH barriers that prevented linkage to care. All identified patients were non-Hispanic Black males with an average age of 37.

Each patient had either a mental health or substance use disorder, with three of the four patients having both, in addition to at least one SDoH barrier. Despite this, our EDs used both ED HIV program staff and other ED wrap-around services (including social work, case management, and financial aid counselors) to successfully link these patients to HIV care. Viral suppression was confirmed in three of the four patients.

While prior case series surrounding ED patients with HIV highlight specific comorbid diseases including COVID-19, pediatric arthropathy, horizontal HIV transmission, encephalitis, and others,^4^–^8^ literature is sparse characterizing the prevalence or discussion of comorbid mental health and substance use disorders, and other SDoH.^2^ This is despite a surge in HIV infections as part of a parallel outbreaks in intravenous drug use.^9^–^14^ In only one study evaluating HIV screening in a Midwestern urban ED was problematic alcohol or other substance use and mental health disorders reported in 58.5% and 47% of patients, respectively.^2^

A brief internal analysis of these two study sites demonstrated that at Site 1 over 60% of patients with HIV had a mental health or substance use disorder, over 40% reported unstable housing, and over 25% endorsed a lack of reliable contact information with or without current or prior incarceration. At Site 2, over 38% reported some substance use and 21% had unstable housing. The co-localization and intersection of the HIV and opioid epidemics is highlighted by the increasing prevalence of substance use disorder among patients with HIV. Despite identifying this association within regional outbreaks, the long-term HIV care successes and challenges of people who inject drugs, who have concomitant mental health or substance use disorders alongside various SDoH have not been described in any detail.

While many articles have cited the SDoH barriers that plague overall ED patient populations, few articles highlight specific barriers in patients with HIV who seek care from the ED.^15^ The HIV epidemic is far from over, and the coexisting opioid epidemic only complicates the issue. This case series highlights several unique cases that discuss at least one SDoH critically impeding successful linkage. We also describe the intersection of other mental health and substance use disorders that affect patients’ ability to initiate HIV treatment. This case series highlights some of our self-identified “toughest cases” to demonstrate how these barriers were mitigated to provide successful linkage to care.

## CONCLUSION

Despite challenging mental health and substance use disorders, and SDoH barriers, these ED-identified, patients with HIV were notified of their disease status and successfully linked to care. The patient navigators used perseverance, connections to local community resources, and leveraged family support to achieve linkage success. As we better understand the full scope of these co-localized patient factors, we will gain a better understanding of the necessary resources for successful linkage and treatment. A deeper understanding of the interaction of HIV with substance use and mental health disorders and SDoH must be prioritized to better facilitate linkage for our most medically and socially complex patients.

## Figures and Tables

**Table:** Summary of demographic, HIV testing, medical comorbidities, social determinants of health, and linkage-to-care efforts.

	Patient 1	Patient 2	Patient 3	Patient 4
**Demographics**				
Age	36	21	35	33
Sex	Male	Male	Male	Male
Race/Ethnicity	Non-Hispanic Black	Non-Hispanic Black	Non-Hispanic Black	Non-Hispanic Black
**Days to Treatment**				
Number of ED visits 12 months prior to HIV screening test	8	0	0	1
Days from screening to confirmatory test	0	0	0	0
Days from confirmatory test to RNA	28	15	1	15
Days from screening to appointment for HIV treatment	47	20	77	0
Days from screening to HIV ART start	33	20	77	0
Days from screening to HIV viral suppression	169	140	168	135
**Medical comorbidities**				
Mental Health	Schizophrenia	Depression, anxiety, post-traumatic stress disorder	Schizophrenia, mood disorder, substance-induced psychosis	ADHD, anxiety, bipolar 1 disorder, depression, schizophrenia
Substance Use Disorder	Alcohol, cocaine	None	Methamphetamine	Tobacco, marijuana
**SDoH variables**				
Transportation	Yes	Yes	No	Yes
Lack of contact information	Yes	No	No	Yes
Incarceration	Yes	Yes	Yes	Yes
Housing insecurity	Yes	Yes	Yes	Yes
Food insecurity	No	Yes	No	Yes

Abbreviations: *ADHD*, attention-deficit/hyperactivity disorder; *ART*, antiretroviral therapy; *ED*, emergency department; *SDoH*, social determinants of health
